# The Evaluation of the Antioxidant and Intestinal Protective Effects of Baicalin-Copper in Deoxynivalenol-Challenged Piglets

**DOI:** 10.1155/2020/5363546

**Published:** 2020-01-22

**Authors:** Andong Zha, Daixiu Yuan, Zhijuan Cui, Ming Qi, Simeng Liao, Peng Liao, Bie Tan

**Affiliations:** ^1^Institute of Subtropical Agriculture, Chinese Academy of Sciences, Changsha, Hunan 410125, China; ^2^University of Chinese Academy of Sciences, Beijing 100008, China; ^3^Department of Medicine, Jishou University, Jishou, Hunan 416000, China

## Abstract

The present study was performed to evaluate the antioxidant and intestinal protective effects of baicalin-copper on deoxynivalenol-challenged piglets. Forty weaned piglets were randomly divided into four groups and assigned to different diets: (1) basal diet (Con), (2) 4 mg/kg deoxynivalenol of basal diet (DON), (3) 5 g/kg baicalin-copper of basal diet (BCU); and (4) 4 mg/kg deoxynivalenol + 5 g/kg baicalin‐copper of basal diet (DBCU). The results showed that the ADFI and ADG of piglets in the DON group were markedly lower than those in the Con group, but the ADFI and ADG of the DBCU group were not significantly different from those of the Con group. In piglets fed a DON-contaminated diet, dietary supplementation with BCU significantly decreased the mRNA levels of P70S6K, 4E-BP1, and HSP70 in the liver, the protein expression of HO-1 in the jejunum, and the expression of p-Nrf2 and p-NF-*κ*B in the ileum but increased Mn-SOD activity in serum. Dietary supplementation with BCU increased jejunal maltase, ZIP4 and MT mRNA levels, and serum concentrations of Arg, Val, Ile, Leu, Lys, and Tyr in DON-contaminated piglets. In summary, BCU can alleviate the growth impairment induced by DON and enhance antioxidant capacity and nutrition absorption in piglets fed DON-contaminated diets.

## 1. Introduction

Deoxynivalenol (DON) is a trichothecene mycotoxin that is mainly produced by *Fusarium graminearum* and *Fusarium culmorum* and is named for its ability to cause nausea and vomiting in animals [1, 2]. DON pollution is common worldwide, and DON has the highest detection rate and overstandard rate among all mycotoxins [3–6]. DON promotes oxidative stress, changes intestinal morphology, and affects nutrient absorption. Additionally, pigs fed a DON-contaminated diet can show growth retardation, weight loss, anorexia, and other toxicity symptoms [7–9]. Some studies have also noted that DON can affect the body's immune regulation mechanism and that DON can induce the activation of the NF-*κ*B pathway and promote the upregulation of a series of cytokines at the protein and mRNA levels. In addition, DON has reproductive genotoxicity, which can reduce fetal quality and increase fetal mortality. A previous study showed that DON can cause morphological damage in intestinal villi epithelial cells in pigs, resulting in the reduced intake or even refusal of food and damaging antioxidant capacity [10, 11].

Baicalin-copper (BCU) is a complex of baicalin and copper ions. Baicalin is extracted from *Scutellaria baicalensis*, and it is a flavonoid. Baicalin can clear free radicals to avoid oxidative damage, has anti-inflammatory and anticancer effects, and promotes cellular and humoral immunity [12–14]. A previous study showed that baicalin easily chelates with Zn^2+^ or Cu^2+^ to form a metal chelate compound, which has a stronger oxygen free radical-scavenging effect than does the baicalin monomer [15]. Li et al. showed that baicalin-copper induces apoptosis in HepG2 cells by downregulating the PI3K/Akt/mTOR signaling pathway and that baicalin-copper reduced the liver relative index, increasing the SOD content in the liver [16]. In an acute toxicity test, Liu et al. showed that BCU not only is nontoxic to mice but also has good antibacterial and antitumor activity [17].

Based on the function of BCU, we hypothesize that the addition of BCU to the diet can alleviate intestinal damage, promote antioxidant capacity, and improve growth performance. The objective of this experiment was to investigate the effects of BCU on intestinal antioxidative damage using a piglet model of DON-induced oxidative stress.

## 2. Materials and Methods

### 2.1. Preparation of DON-Contaminated Feed and BCU

The DON-contaminated diet was prepared according to previous studies [9, 18]. The contents of mycotoxins in the diet were detected by liquid chromatography (Beijing Taileqi, Qingdao, China).  Table 1 shows the toxin content in the basal diet: the DON content in the basal diet was 124 *μ*g/kg, the DON content in the DON-contaminated diet was 7964 *μ*g/kg, and the AFB_1_ and ZEN contents were low in the basal diet and DON-contaminated diet. Before the experiment, basal diet was added to the DON-contaminated diet to reach a DON content of 4 mg/kg diet. The concentration of DON was chosen according to previous studies [19, 20]. The synthesis of BCU was based on a previous study [16, 17]. The dose of BZN used in the present experiment was based on a preliminary test.

### 2.2. Animals, Diets, and Experimental Design

Our study included 40 weaned piglets (21 d) as experimental animals. All piglets were provided by New Wellful (New Wellful Co. Ltd., Changsha, China). Forty pigs were randomly divided into 4 groups with 10 repeats in each group and fed different diets: (1) basal diet (Con group), (2) 4 mg/kg deoxynivalenol of basal diet (DON group), (3) 5 g/kg baicalin-copper of basal diet (BCU group), and (4) 4 mg/kg deoxynivalenol + 5 g/kg baicalin‐copper of basal diet (DBCU group). The basal diet was a corn-soybean mash diet. According to NRC (2012), weaned piglets' nutritional needs and the ideal amino acid model preparation for pigs, the basic diet composition, and the nutrient level are shown in Table 2 [21]. The experimental design and all procedures involving animals were approved by the Animal Care and Use Committee of the Institute of Subtropical Agriculture at the Chinese Academy of Sciences.

### 2.3. Animal Management and Sample Collection

The piglets were housed individually in stalls with a slatted floor, and the room temperature was maintained at approximately 30°C. The piglets had free access to food and water. The piglets were acclimated to the basal diet for three days before beginning the experiment. During the experiment, the piglets were fed 4 times per day, and the feeding times were 7:30, 11:00, 14:30, and 18:00. The experiment lasted for 14 days [8, 22]. During the trial, one piglet in the Con group died. On the morning of the 15^th^ day, we calculated the growth performance index and randomly selected 7 piglets from each group to slaughter. The samples were stored at -80°C. The following growth performance equations were used:

(1) $$\begin{array}{c} \text{ADG}=\left(\text{final weight}-\text{initial weight}\right)/\text{experimental period}\left(\text{g}/\text{d}\right),\\ \text{ADFI}=\text{total feed intake}/\text{experimental period }\left(\text{g}/\text{d}\right),\\ \text{F}/\text{D}=\text{total feed intake}/\left(\text{final weight}-\text{initial weight}\right),\\ \text{Relative organ weight }\left(\%\right)=\left(\text{organ weight}/\text{body weight}\right)\times 100. \end{array}$$

### 2.4. Intestinal Morphology

First, 2 cm of the jejunum was collected, the chyme was washed, and the sample was fixed in a 4% paraformaldehyde solution. Then, the sample was dehydrated with gradient alcohol solutions and embedded in paraffin. Second, 5 mm slices were prepared and stained with hematoxylin-eosin. Third, microscopes were used to observe the samples, and computer-aided systems were used to photograph the samples. Finally, an image analysis system was used to measure villi length and crypt length [23, 24].

### 2.5. Determination of Antioxidant Capacity and Free Amino Acids in the Serum and Digestive Enzyme Activity in the Intestine

Blood samples were naturally placed at 4°C for 1 hour and centrifuged for 10 minutes (3000 rpm), and the serum was collected. The samples were analyzed in the Laboratory of Animal and Molecular Nutrition of the Institute of Subtropical Agriculture, Chinese Academy of Sciences. The experiments were performed according to the instructions of the ELISA kit (Nanjing Jiancheng Bioengineering Institute, Nanjing, China). The indexes of the serum antioxidant capacity included T-SOD (U/ml), CuZn-SOD (U/ml), Mn-SOD (U/ml), GSH-P*x* (U/ml), T-AOC (mmol/gprot), GSH (U/ml), MDA (nmol/ml), and NO (*μ*mol/l) [25–27]. The indexes of the chyme digestive enzyme activity included trypsin, lipase, maltase, sucrase, and total protein.

Serum free amino acid content was determined using an Ultimate 3000 HPLC and AB 3200 Q TRAP LC-MS/MS. The indexes included L-histidine (His), L-serine (Ser), L-arginine (Arg), glutamic acid or glutamine (Glx), L-aspartic acid or L-asparagine (Asx), L-glutamate (Glu), L-threonine (Thr), L-alanine (Ala), L-proline (Pro), L-cystine (Cys), L-lysine (Lys), L-tyrosine (Tyr), L-methionine (Met), L-valine (Val), L-leucine (Leu), L-isoleucine (Ile) [28, 29].

### 2.6. Quantification of mRNA by Real-Time PCR Analysis

According to previous studies, the tissues were milled in liquid nitrogen, and RNA was extracted by the TRIzol method. Based on the cDNA sequence of pigs, primers were designed by Primer 5.0 (Table 3). Real-time polymerase chain reaction (PCR) was performed, and the results were calculated as previously described [24, 30].

### 2.7. Western Blotting

According to the instructions of the BCA protein assay kit (Wellbio, Changsha, China), we measured the protein concentration in the experiment. The protein was separated by a 4.8% SDS-PAGE stacking gel and a 10% SDS-PAGE separating gel. The protein was transferred to an NC membrane and blocked with 5% skim milk powder. Then, suitable primary and secondary antibodies were used (as in Table 4). Ultimately, an enhanced chemiluminescence (ECL) solution (Thermo Fisher Scientific, Waltham, USA) was used for color exposure, and Image-Pro Plus software was used for protein grayscale analysis [10, 31].

### 2.8. Statistical Analysis

All data were sorted out by Excel (Microsoft, Redmond, USA), one-way ANOVA was conducted with SPSS 20 (IBM, Armonk, USA), and multiple comparisons were performed with Duncan's multiple comparisons. The data of each group were expressed as the mean ± SEM; the significant difference criterion was *p* < 0.05. All figures were prepared with GraphPad Prism 7.0 (GraphPad Software, San Diego, USA).

## 3. Results

### 3.1. Growth Performance and Relative Organ Weights

As shown in Table 5, there was no marked difference in initial weight of the four groups. The final weight, ADFI, and ADG of the DON group were significantly lower than those of the Con group (*p* < 0.05). Compared with the DON group and Con group, the DBCU group did not show marked changes in final weight, ADFI, and ADG. There was no significant difference between the Con group and the BCU group. In addition, no differences in F/G were detected among the four groups.

As shown in Table 6, there was no remarkable difference between the Con group and the BCU group in the relative organ weights of the heart, lung, and kidney. However, the relative weight of the liver in the DBCU group was significantly lower than that in the Con, DON, and BCU groups (*p* < 0.05). In addition, the relative spleen weight was decreased in piglets fed a DON-supplemented diet compared with piglets in the Con and BCU groups (*p* < 0.05), whereas there was no significant difference among the Con, BCU, and DBCU groups.

### 3.2. Serum Antioxidant Capacity

**Figure 1:**
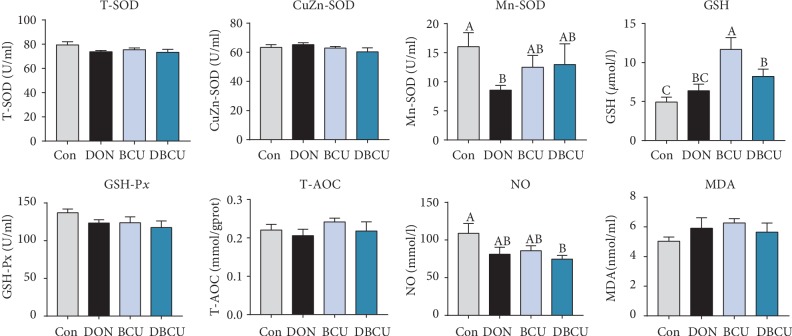
Effects of BCU on the serum antioxidant capacity of piglets challenged with DON (*n* = 7). Data are presented as mean ± SEM; ^A,B^used to indicate a statistically significant difference (*p* < 0.05). Con: basal diet; DON: 4 mg/kg deoxynivalenol of basal diet; BCU: 5 g/kg baicalin-copper of basal diet; DBCU: 4 mg/kg deoxynivalenol + 5 g/kg baicalin‐copper of basal diet.

As Figure 1 shows, although there was no difference in T-SOD activity among the four groups, the Mn-SOD activity in the DON group was significantly lower than that in the Con group (*p* < 0.05), and the Mn-SOD activity in the DBCU group was not different from that in the Con group. The GSH activity of piglets fed BCU was increased compared with that of piglets in the Con group (*p* < 0.05). There was no difference in GSH activity between the DON group and the Con group, but the GSH activity of the DBCU group was significantly higher than that of the Con group (*p* < 0.05). The NO content of the DON group and BCU group was not significantly different from that of the Con group, but the NO content of the DBCU group was significantly lower than that of the Con group (*p* < 0.05).

### 3.3. Intestinal Morphology

**Figure 2:**
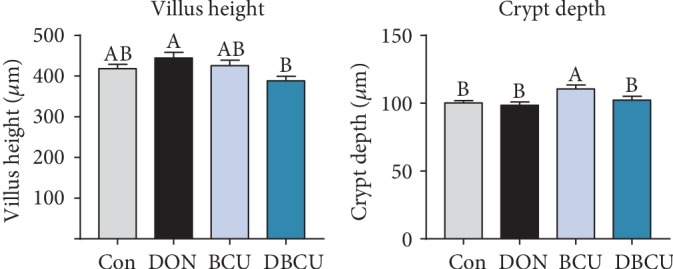
Effects of BCU on the jejunal morphology of piglets challenged with DON (*n* = 7). Data are presented as mean ± SEM; ^A,B^used to indicate a statistically significant difference (*p* < 0.05). Con: basal diet; DON: 4 mg/kg deoxynivalenol of basal diet; BCU: 5 g/kg baicalin-copper of basal diet; DBCU: 4 mg/kg deoxynivalenol + 5 g/kg baicalin‐copper of basal diet.

As illustrated in Figure 2, the jejunum villus height was not decreased in the DON and BCU groups compared with the Con group, but the jejunum villus height of the DBCU group was significantly reduced compared with that of the Con group (*p* < 0.05). There was no remarkable difference in crypt depth among the Con, DON, and DBCU groups, while the piglets fed the BCU-supplemented diet had a markedly increased crypt depth in the jejunum (*p* < 0.05).

### 3.4. Expression of Oxidative Stress-Related Genes in the Liver

**Figure 3:**
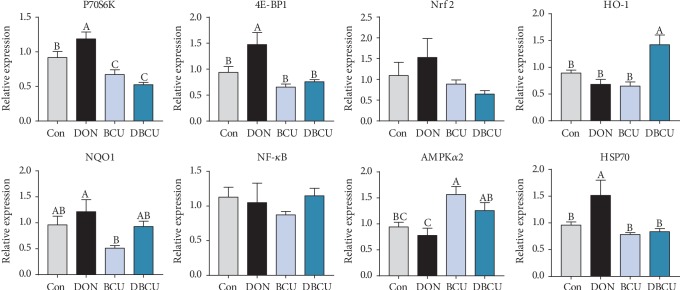
Effects of BCU on oxidative stress-related genes in the liver of piglets challenged with DON (*n* = 7). Data are presented as mean ± SEM; ^A–C^used to indicate a statistically significant difference (*p* < 0.05). Con: basal diet; DON: 4 mg/kg deoxynivalenol of basal diet; BCU: 5 g/kg baicalin-copper of basal diet; DBCU: 4 mg/kg deoxynivalenol + 5 g/kg baicalin‐copper of basal diet.

DON can cause oxidative stress in the body, and the liver is an organ with antioxidant and detoxifying functions, so we tested the mRNA expression of antioxidant genes in the liver. As shown in Figure 3, P70S6K, 4E-BP1, and HSP70 mRNA expression levels were markedly increased in piglets fed the DON-supplemented diet compared with piglet in the Con group (*p* < 0.05); however, the P70S6K mRNA expression in the DBCU group was remarkably lower than that in the Con group (*p* < 0.05). The 4E-BP1 and HSP70 mRNA expression levels in the DBCU group were not different from those in the Con group. In addition, the HO-1 mRNA expression of the DBCU group was significantly higher than that in the Con group (*p* < 0.05). The P70S6K mRNA expression of the BCU group was lower than that of the Con group, and AMPK*α*2 mRNA expression was higher than that in the Con group (*p* < 0.05). Compared with the DBCU group, the P70S6K, 4E-BP1, and HSP70 mRNA expression levels in the DON group were increased (*p* < 0.05), and the HO-1 and AMPK*α*2 mRNA expression levels in the DON group were decreased (*p* < 0.05).

### 3.5. Western Blotting of Intestinal Samples

**Figure 4:**
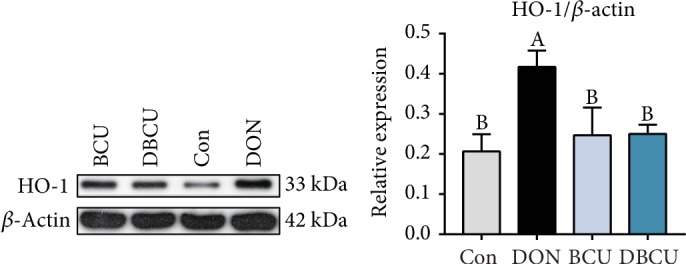
Effects of BCU on the relative protein level in the jejunum of piglets challenged with DON. Data are presented as mean ± SEM; ^A–C^used to indicate a statistically significant difference (*p* < 0.05). Con: basal diet; DON: 4 mg/kg deoxynivalenol of basal diet; BCU: 5 g/kg baicalin-copper of basal diet; DBCU: 4 mg/kg deoxynivalenol + 5 g/kg baicalin‐copper of basal diet.

**Figure 5:**
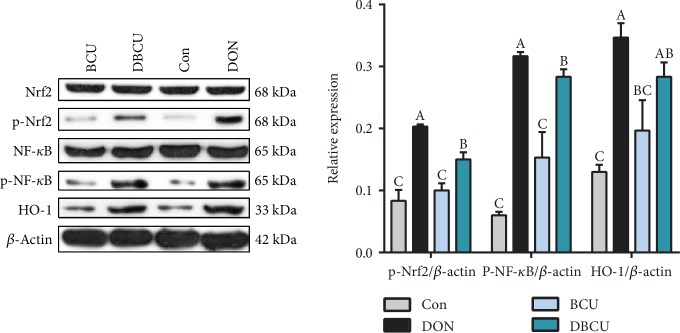
Effects of BCU on the relative protein level in the ileum of piglets challenged with DON. Data are presented as mean ± SEM; ^A–C^used to indicate a statistically significant difference (*p* < 0.01). Con: basal diet; DON: 4 mg/kg deoxynivalenol of basal diet; BCU: 5 g/kg baicalin-copper of basal diet; DBCU: 4 mg/kg deoxynivalenol + 5 g/kg baicalin‐copper of basal diet.

DON is mainly absorbed in the intestine, so we investigated whether DON affects the expression of antioxidant proteins. As shown in Figure 4, in the jejunum, compared with piglets in the Con, BCU, and DBCU groups, the piglets in the DON group showed markedly increased HO-1 expression. As shown in Figure 5, in the ileum, compared with piglets in the Con group, piglets of the DON group showed markedly increased protein expression levels of p-Nrf2, p-NF-*κ*B, and HO-1 (*p* < 0.05), while piglets fed the DON and BCU supplemented diet showed decreased p-Nrf2 and p-NF-*κ*B expression levels in compare with DON group (*p* < 0.05). There was no difference in p-Nrf2, p-NF-*κ*B, and HO-1 expression between the Con and BZN groups.

### 3.6. Serum Free Amino Acids

**Figure 6:**
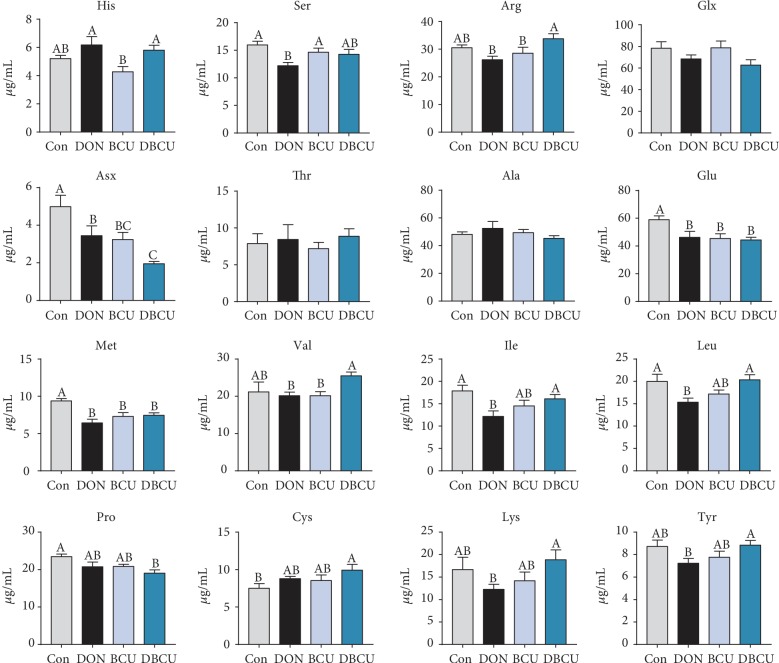
Effects of BCU on the free amino acids in the serum of piglets challenged with DON. Data are presented as mean ± SEM; ^A–C^used to indicate a statistically significant difference (*p* < 0.05). Con: basal diet; DON: 4 mg/kg deoxynivalenol of basal diet; BCU: 5 g/kg baicalin-copper of basal diet, DBCU: 4 mg/kg deoxynivalenol + 5 g/kg baicalin‐copper of basal diet.

Figure 6 shows the results. Piglets fed the DON-supplemented diet showed significantly decreased Ser, Asx, Glu, Met, Ile, and Leu content in serum (*p* < 0.05), while piglets fed the BCU-supplemented diet showed significantly decreased Asx, Glu, Met, and Pro content (*p* < 0.05). Compared with the DON group, the DBCU group had obviously decreased Asx content and the DBCU group had clearly enhanced Ile, Arg, Leu, Tyr, and Lys content (*p* < 0.05).

### 3.7. Intestinal Nutrition Digestive and Absorption-Related Gene Expressions

**Figure 7:**
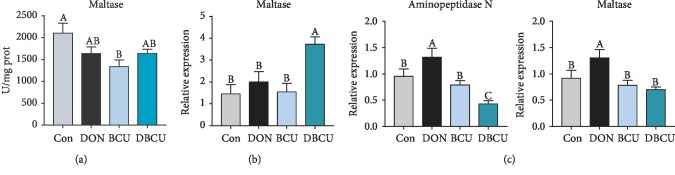
Effects of BCU on digestive enzyme-related genes in the intestine of piglets challenged with DON (*n* = 7). (a) Chyme maltase enzyme; (b) jejunum; (c) ileum. Data are presented as mean ± SEM; ^A–C^used to indicate a statistically significant difference (*p* < 0.05). Con: basal diet; DON: 4 mg/kg deoxynivalenol of basal diet; BCU: 5 g/kg baicalin-copper of basal diet; DBCU: 4 mg/kg deoxynivalenol + 5 g/kg baicalin‐copper of basal diet.

As shown in Figure 7, compared with the control diet, dietary supplementation with BCU at 5 g/kg diet reduced the maltase activity in the chyme (*p* < 0.05), but there was no evident difference in maltase activity between the BCU and DBCU groups. In the jejunum, the maltase mRNA levels of the DBCU group were significantly higher than that of the other groups (*p* < 0.05). In the ileum, the aminopeptidase N and maltase mRNA levels in the DON group were significantly higher than those in the Con group (*p* < 0.05), the aminopeptidase N mRNA levels in the DBCU group were significantly lower than those in the Con group and the DON group (*p* < 0.05). The aminopeptidase N and maltase mRNA levels in the BCU group were not significantly different from those in the Con group.

**Figure 8:**
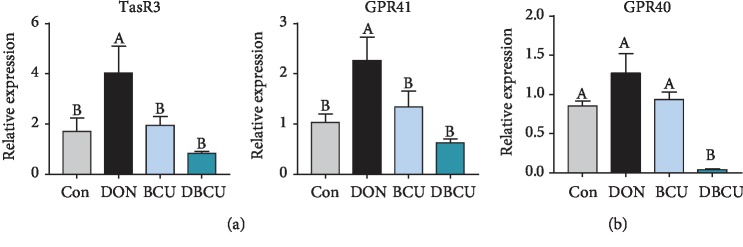
Effects of BCU on nutrient-sensing genes in the intestine of piglets challenged with DON (*n* = 7). (a) Jejunum; (b) ileum. Data are presented as mean ± SEM; ^A–C^used to indicate a statistically significant difference (*p* < 0.05). Con: basal diet; DON: 4 mg/kg deoxynivalenol of basal diet; BCU: 5 g/kg baicalin-copper of basal diet; DBCU: 4 mg/kg deoxynivalenol + 5 g/kg baicalin‐copper of basal diet.

As shown in Figure 8, in the jejunum, the TasR3 and GPR41 mRNA levels in the DON group were significantly higher than those in the other three groups (*p* < 0.05), while there was no significant difference in TasR3 and GPR41 mRNA levels among the Con, BCU, and DBCU groups. In the ileum, no differences in GPR40 mRNA expression were detected compared with the Con group, while GPR40 mRNA levels in DBCU group were significantly reduced (*p* < 0.05).

**Figure 9:**
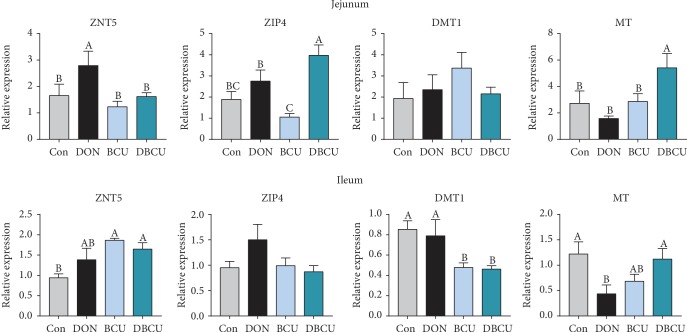
Effects of BCU on zinc transporter genes in the intestine of piglets challenged with DON (*n* = 7). Data are presented as mean ± SEM; ^A,B^used to indicate a statistically significant difference (*p* < 0.05). Con: basal diet; DON: 4 mg/kg deoxynivalenol of basal diet; BCU: 5 g/kg baicalin-copper of basal diet; DBCU: 4 mg/kg deoxynivalenol + 5 g/kg baicalin‐copper of basal diet.

As Figure 9 shows, in the jejunum, the mRNA level of ZNT5 in the DON group was significantly higher than that in the Con group (*p* < 0.05). The ZNT5 mRNA level in the DBCU group was significantly lower than that in the DON group (*p* < 0.05), while the ZIP4 and MT mRNA levels in the DBCU group were significantly higher than those in the DON group (*p* < 0.05). In the ileum, the MT mRNA level in the DON group was significantly lower than that in the Con group. The ZNT5 mRNA level in the DBCU group was significantly higher than that in the Con group, the MT mRNA level in the DBCU group was significantly higher than that in the DON group, and the DMT1 mRNA level in the DBCU group was significantly lower than that in the Con group and the DON group. The ZNT5 mRNA level in the BCU group was significantly higher than that in the Con group, and the DMT1 mRNA level in the BCU group was significantly lower than that in the Con group.

## 4. Discussion

The degree of DON contamination in grain is the highest among fusarium toxins and poses a large threat to animal health. Previous studies showed that deoxynivalenol reduced or mutilated feeding and caused vomiting and weight loss. Xiao et al. found that DON significantly reduced ADG and ADFI in weaned piglets through a 30-day feeding trial [8]. Li et al. also showed that piglets fed a DON-contaminated diet showed significantly decreased growth performance [32]. Goyarts et al. found that DON did not change the feed-to-gain ratio of grower pigs, while Bergsjo et al. showed that grower piglets fed DON-contaminated diets had reduced feed utilization efficiency [33, 34]. Baicalin is a kind of flavonoid compound. Some studies have also shown that dietary flavonoid supplementation can ameliorate oxidative stress and improve the growth performance of broilers and pigs [35–38]. The results of the current study show that dietary supplementation with DON at 4 mg/kg diet significantly decreased ADFI and ADG, and did not impact F/G. However, dietary supplementation with BCU and DON did not change the ADFI and ADG in compare with Con group, which means that dietary supplementation with BCU can improve growth performance in deoxynivalenol-challenged piglets. It has also been shown that oral DON administration in mice causes a hepatic inflammatory response and spleen immune dysfunction [39, 40]. Different studies have shown different effects of DON on relative organ weight [41, 42]. In the current study, the relative liver weight of the DBCU group was obviously decreased compared with that of the DON group, and the relative spleen weight of the DBCU group tended to be higher than that of the DON group. This result may be attributed to the protective effect of baicalin on the liver and spleen, as previous studies showed [16, 43, 44].

Previous studies have suggested a toxicological mechanism of deoxynivalenol: DON binds with the 80S subunit on the ribosome in eukaryotic cells, activates the MAPK pathway, produces an oxidative stress response, inhibits the synthesis of DNA and proteins, and thus produces cytotoxicity and immunotoxicity [45–48]. In contrast, baicalin, a kind of flavonoid compound, has a good antioxidant effect. Previous studies have reported that, on the one hand, flavonoids can affect enzyme activity, including enhancing antioxidant activity and inhibiting oxidase activity; on the other hand, flavonoids directly remove reactive oxygen species [49–52]. To understand the effects of DON and BCU on the antioxidant capacity of piglets, we first measured the serum antioxidant index of piglets. In the current study, dietary supplementation with DON reduced Mn-SOD activity in serum and BCU partly alleviated the effect of DON on Mn-SOD activity. Dietary supplementation with DON and BCU markedly reduced the concentration of NO in serum. GSH is an important antioxidant that can scavenge free radicals in the body. Dietary supplementation with BCU increases the concentration of GSH in serum. Yang et al. and Calabrese et al. showed that the addition of DON to feed resulted in oxidative stress in the jejunum of broiler chickens [53, 54]. Therefore, we further measured the expression of intestinal antioxidant proteins. In the present study, dietary supplementation with DON markedly increased HO-1 protein expression in the jejunum and HO-1, p-Nrf2, and p-NF-*κ*B protein expression in the ileum. Compared with DON supplementation, dietary supplementation with DON and BCU markedly decreased HO-1 protein expression in the jejunum and p-Nrf2, and p-NF-*κ*B contents in the ileum. These results indicate that dietary supplementation with BCU at 5 g/kg diet can inhibit the expression of intestinal oxidative stress-related proteins in deoxynivalenol-challenged pigs. These results were similar to those of a previous study, which found that flavonoids activate antioxidant capacity and alleviate intestinal injury through the NF-*κ*B and Nrf2 pathway [55–57]. The liver is an important organ for the detoxification of the body and has good antioxidant activities. Therefore, we detected the expression of antioxidant genes in the liver. Dietary supplementation with DON also markedly increased the mRNA levels of P70S6K, 4E-BP1, and HSP70 in the liver. Compared with DON supplementation, dietary supplementation with DON and BCU obviously reduced the mRNA levels of P70S6K, 4E-BP1, and HSP70 and increased the mRNA levels of AMPK*α*2 and HO-1 in the liver. These results indicate that BCU can alleviate oxidative stress in the liver of DON-challenged pigs. Some articles have also reported that flavonoids activate the AMPK pathway, inhibit the expression of Nrf2 and HO-1, and protect the liver in mice [44, 58, 59]. In conclusion, according to the intestinal, liver, and serum results, dietary supplementation with BCU improved the antioxidant capacity of piglets, thus alleviating the damage caused by DON.

It can be seen from the above findings that dietary supplementation with DON reduces growth performance and causes oxidative stress damage in piglets. The intestine is the primary target after DON enters the body. The decline in growth performance has a negligible link with intestinal damage by DON. Previous studies showed that DON can induce intestinal epithelial cell injury, change villus height and crypt depth in the intestine, and block the absorption of nutrients [1, 60, 61]. In the present study, the villus height in the DON group was significantly higher than that in the DBCU group, which was different from a previous study [10]. The digestion and absorption of nutrients in the intestine requires digestive enzymes. In the chyme, the maltase activity of the BCU group decreased. In the jejunum, the maltase mRNA levels of the DBCU group were significantly higher than those of the other three groups. In the ileum, the levels of aminopeptidase N and maltase mRNA of the DON group were significantly higher than those of the other three groups. This result may be because maltase in the DON group was damaged, so more maltase was produced for digestion. The absorption of nutrients by the intestines begins with nutrient-sensing genes. In the jejunum, TasR3 and GPR41 mRNA levels were significantly increased in the DON group, possibly because the DON group was nutrient deficient and thus expressed more receptors to absorb nutrients. Amino acids are the energy source of piglets, which are transported to various tissues with the blood. To understand the amino acid metabolism of piglets, we also measured the content of free amino acids in the blood. Lysine is the first restricted amino acid in pigs. The lack of lysine can lead to decreased growth performance in pigs. The serum lysine content in the DBCU group was significantly higher than that in the DON group, which means that BCU may increase the absorption of Lys in DON-challenged piglets. Phosphorylation is a common posttranslational modification that plays an important role in regulating signal transduction and gene expression [62, 63]. In eukaryotes, phosphorylation mainly occurs on serine, threonine, and tyrosine residues [28, 62]. The current study showed that the Ser concentration of the DON group tended to be lower than that of the DBCU group. This result may be because DON promotes the phosphorylation of antioxidant-related proteins and consumes a large amount of serine, and dietary supplementation with DON and BCU can reduce the phosphorylation of antioxidant-related proteins. In addition, the Leu concentration of the DON group was also markedly lower than that of the Con and DBCU groups. Leucine is not only a substrate for protein synthesis but also a key regulatory factor for protein synthesis in muscle tissues. The declined growth performance of the DON group may also be related to the blocked absorption of Leu [64, 65].

## 5. Conclusions

In summary, dietary BCU plays a beneficial role in alleviating the effect of DON on piglets, such as improving growth performance and antioxidant capacity and regulating intestinal nutrition absorption in weaned piglets. The results improve our understanding of the function of BCU in the intestine and support the application of BCU in animal production.

## Figures and Tables

**Table 1:** Mycotoxin content in contaminated feed (*n* = 6).

Items	AFB_1_ (*μ*g/kg)	DON (*μ*g/kg)	ZEN (*μ*g/kg)
Limit of detection	1	100	10
Basal feed	Undetected	124	53
DON-contaminated feed	7.3	7964	12.56

**Table 2:** Composition and nutrient level of the diets.

Ingredients	Content (%)	Nutrient levels	Content
Corn	63.70	DE (MJ/kg)	14.60
Soybean meal	19.80	CP (%)	20.27
Whey dried	4.30	Calcium (%)	0.69
Fish meal	9.00	Phosphorus (%)	0.57
Soybean oil	0.80	Lysine (%)	1.26
Lysine	0.38	Threonine (%)	0.76
Methionine	0.10	Met+Cys (%)	0.62
Threonine	0.09	Tryptophan (%)	0.20
Tryptophan	0.01	Arginine (%)	1.09
Limestone	0.52	Histidine (%)	0.44
NaCl	0.30	Isoleucine (%)	0.71
Premix^∗^	1.00	Leucine (%)	1.52
		Phenylalanine (%)	0.81
		Valine (%)	0.72

^∗^The premix feed contain Cu 5 mg, Se 0.3 mg, I 0.1 mg, Fe 80 mg, Zn 85 mg, Mn 3 mg, vitamin B_1_ 1 mg, vitamin B_2_ 3 mg, vitamin B_3_ 12.5 mg, vitamin B_6_ 1.6 mg, vitamin B_5_ 10 mg, vitamin A 2000 IU, vitamin B_12_ 0.016 mg, vitamin D_3_ 200 IU, vitamin E 12 IU, vitamin K 0.5 mg, folic acid 0.3 mg, choline chloride 0.5 mg, vitamin B_7_ 0.05 mg.

**Table 3:** Primers used for RT-PCR.

Gene	Primer sequence (5′-3′)	Accession number	Size (bp)	Tm (°C)
Nrf2	CACCACCTCAGGGTAATA	XM_021075133.1	125	60
	GCGGCTTGAATGTTTGTC			
HO-1	AGCTGTTTCTGAGCCTCCAA	NM_001004027.1	130	60
	CAAGACGGAAACACGAGACA			
NQO1	CCAGCAGCCCGGCCAATCTG	NM_001159613.1	160	60
	AGGTCCGACACGGCGACCTC			
NF-*κ*B	AGCCATTGACGTGATCCAGG	NM_001048232.1	248	60
	CGAAATCGTGGGGCACTTTG			
AMPK*α*2	CGACGTGGAGCTGTACTGCTT	XM_021091195.1	143	60
	CATAGGTCAGGCAGAACTTGC			
P70S6K	GGAAACAAGTGGAATAGAGCAGATG	XM_021067294.1	65	60
	TTGGAAGTGGTGCAGAAGCTT			
4E-BP1	CCGGAAGTTCCTAATGGAGTGT	NM_001244225.1	125	60
	GGTTCTGGCTGGCATCTGT			
HSP70	GTGGCTCTACCCGCATCCC	NM_001123127.1	114	60
	GCACAGCAGCACCATAGGC			
Aminopeptidase N	TCATCAATCGGGCTCAGGTC	XM_005653524.3	101	61
	TCCGTTCAGGAAGAGGGTGTT			
Maltase	TGCCTTACCTCTACACGCTGATGC	XM_021079095.1	232	65
	GATTCACTGCCAGATTCCGTGCTAT			
ZnT1	CCAGGGGAGCAGGGAACCGA	NM_001139470.1	73	65
	TCAGCCCGTTGGAGTTGCTGC			
ZnT2	GACAGCGCCAGCCAGCATCA	NM_001139475.1	99	65
	GGCAGCCACCAAAACGCCCA			
ZnT5	ACCAGTCTCAGTTGGAGGGCTGA	NM_001137624.1	79	65
	TCCATGGGTATGGGTGTGGGCA			
ZIP4	TGCTGAACTTGGCATCTGGG	XM_021090449.1	125	60
	CGCCACGTAGAGAAAGAGGC			
DMT1	CGCGCTTCGCCCGAGTGAT	XM_021081710.1	70	66
	TGGAAGACGGCCACCAGCAGA			
MT	GTGAATCCGCGTTGCTCTCTGCT	XM_021093891.1	72	66
	CTGTGGGGCAGGAGCAGTTGG			
TasR3	GCTGGGCGACAGGACAG	NM_001113288.1	102	60
	TTGATTTCCTCCACAGCCAT			
GPR40	TGCTCTGACCTCCTGCTGG	XM_013998289.2	235	60
	CACACACCCCCCAGGAATAG			
GPR43	CGTGTTCATCGTTCAGTA	XM_021093196.1	76	52
	GAAGTTCTCATAGCAGGTA			
GAPDH	ACACTCACTCTTCTACCTTTG	XM_021091114.1	90	60
	CAAATTCATTGTCGTACCAG			

**Table 4:** Antibody information used in Western blotting.

First antibody	Company	Catalog number	Titers
P70S6K	Abcam	ab9366	1 : 250
p-P70S6K	LSBio	LS-C124497	1 : 500
HO-1	Abcam	ab52947	1 : 2000
mTOR	Proteintech	20657-1-AP	1 : 300
p-mTOR	Abcam	ab109268	1 : 1000
NF-*κ*B	Proteintech	10745-1-AP	1 : 500
p-NF-*κ*B	Abcam	ab86299	1 : 2000
Nrf2	Abcam	ab92946	1 : 1000
p-Nrf2	Bioss	bs-2013R	1 : 500
*β*-Actin	Proteintech	60008-1-Ig	1 : 5000

**Table 5:** Effects of BCU on growth performance of piglets challenged with DON (*n* = 7).

Item	Con	DON	BCU	DBCU	SEM	*p* value
Initial weight (kg)	6.036	6.007	6.414	6.129	0.13	0.696
Final weight (kg)	9.343^ab^	8.236^b^	9.736^a^	8.786^ab^	0.206	0.044
ADFI (g/d)	315.816^a^	227.551^b^	321.939^a^	277.551^ab^	12.054	0.012
ADG (g/d)	236.226^a^	159.183^b^	237.246^a^	189.796^ab^	9.84	0.004
F/G	1.338	1.434	1.365	1.501	0.031	0.255

^a,b^Means in the same row with different superscripts differ (*p* < 0.05). Con: basal diet; DON: 4 mg/kg deoxynivalenol of basal diet; BCU: 5 g/kg baicalin-copper of basal diet, DBCU: 4 mg/kg deoxynivalenol + 5 g/kg baicalin‐copper of basal diet.

**Table 6:** Effects of BCU on relative organ weights of piglets challenged with DON (*n* = 7).

Item	Con	DON	BCU	DBCU	SEM	*p* value
Heart (%)	0.509	0.486	0.481	0.457	0.469	0.069
Liver (%)	2.530^a^	2.523^a^	2.386^a^	2.166^b^	2.313	0.003
Spleen (%)	0.254^a^	0.186^b^	0.257^a^	0.224^ab^	0.209	0.047
Lung (%)	1.354	1.170	1.266	1.211	1.175	0.330
Kidney (%)	0.639	0.553	0.614	0.551	0.556	0.129

^a,b^Means in the same row with different superscripts differ (*p* < 0.05). Con: basal diet; DON: 4 mg/kg deoxynivalenol of basal diet; BCU: 5 g/kg baicalin-copper of basal diet; DBCU: 4 mg/kg deoxynivalenol + 5 g/kg baicalin‐copper of basal diet.

## Data Availability

All data used to support the findings of this study are included within the article.

## Abbreviations

ADG: Average daily gain
ADFI: Average daily feed intake
AFB_1_: Aflatoxin B_1_
ALP: Alkaline phosphatase
ALT: Alanine aminotransferase
AST: Aspartate aminotransferase
Asx: L-Aspartic acid or L-asparagine
Arg: L-Arginine
Ala: L-Alanine
AMPK*α*2: AMP-activated protein kinase *α* 2
AKT: AKT serine/threonine kinase 1
BCU: Baicalin-copper
BUN: Blood urea nitrogen
CHOL: Cholesterol
CR: Creatinine
CuZn-SOD: CuZn superoxide dismutase
Cys: L-Cystine
DMT1: Divalent metal transporter-1
DON: Deoxynivalenol
EAA: Essential amino acids
F/G: Feed/gain
GLU: Glucose
GSH-P*x*: Glutathione peroxidase
Glx: Glutamic acid or glutamine
Glu: L-Glutamate
GAPDH: Glyceraldehyde-3-phosphate dehydrogenase
GPR40: G protein-coupled receptors 40
GPR41: G protein-coupled receptors 41
His: L-Histidine
HO-1: Heme oxygenase-1
HSP70: Heat shock protein 70
Ile: L-Isoleucine
Lys: L-Lysine
Leu: L-Leucine
Mn-SOD: Mn superoxide dismutase
MDA: Malondialdehyde
Met: L-Methionine
MT: Metallothionein-1
NEAA: Nonessential amino acids
Nrf2: Nuclear factor erythroid-2-related factor 2
NQO1: NAD(P)H quinone oxidoreductase-1
NF-*κ*B: Nuclear factor kappa-B
Pro: L-Proline
Phe: L-Phenylalanine
P70S6K: Phosphorylated 70 kDa ribosomal S6 kinase
Ser: L-Serine
T-AOC: Total antioxidizing capability
T-NOS: Total nitric oxide synthase
T-SOD: Total superoxide dismutase
Thr: L-Threonine
Tyr: L-Tyrosine
Try: L-Tryptophan
TasR3: Taste receptor type 1 member 3
Val: L-Valine
ZEN: Zearalenone
ZNT1: Zinc exporter 1
ZNT2: Zinc exporter 2
ZNT5: Zinc exporter 5
ZIP4: Zinc transporter
4E-BP1: eIF4E-binding protein 1.
